# Prevalence and Clinical Characteristics of Myocardial Infarction With Non-obstructive Coronary Arteries Compared With Obstructive Myocardial Infarction: A Retrospective Tertiary-Centre Study

**DOI:** 10.7759/cureus.117051

**Published:** 2026-09-28

**Authors:** Amina Al Tawil, Rijad Jahić, Alen Džubur, Adnan Al Tawil, Daniela Lončar, Azra Durak Nalbantic

**Affiliations:** 1 Department of Cardiology, Dr. Al Tawil Polyclinic, Sarajevo, BIH; 2 Clinic for Heart, Blood Vessel and Rheumatic Diseases, Clinical Centre University of Sarajevo, Sarajevo, BIH; 3 Department of Radiology, Dr. Al Tawil Polyclinic, Sarajevo, BIH; 4 Clinic for Internal Medicine, University Clinical Centre Tuzla, Tuzla, BIH

**Keywords:** acute coronary syndrome, cardiovascular risk profile, coronarography, echocardiography, non-obstructive coronary disease

## Abstract

Introduction: Myocardial infarction with non-obstructive coronary arteries (MINOCA) is a working diagnosis in patients who fulfil the diagnostic criteria for acute myocardial infarction (AMI) but have no coronary artery stenosis ≥50% and no clinically evident alternative diagnosis that adequately explains the presentation. It encompasses heterogeneous coronary mechanisms.

Objective: To determine the prevalence and clinical characteristics of MINOCA at a tertiary cardiac centre in Bosnia and Herzegovina and to compare demographic characteristics, cardiovascular risk factors, electrocardiographic presentation, laboratory parameters and echocardiographic findings with those of patients with obstructive myocardial infarction (OMI).

Methods: This retrospective single-centre observational study comprised a prevalence analysis and a comparative analysis. For the prevalence analysis, medical records of 2,341 patients admitted to the Intensive Cardiac Care Unit between April 2022 and February 2024 were reviewed, of whom 1,104 fulfilled the diagnostic criteria for AMI. For the comparative analysis, 40 patients with MINOCA identified between January 2020 and February 2024 were compared with a simple random sample of 155 eligible patients with OMI. Demographic characteristics, cardiovascular risk factors, electrocardiographic presentation, laboratory parameters and echocardiographic variables were analysed.

Results: MINOCA accounted for 32 (2.9%) MIs in the prevalence analysis. Patients with MINOCA (N = 40) were younger than patients with OMI (N = 155) (52.1 ± 15.2 vs. 59.8 ± 11.7 years; p = 0.005). Among patients with available sex data, female sex was more frequent in the MINOCA group (19 {47.5%}) than in the OMI group (43 {29.9%}; p = 0.037). The MINOCA group had a shorter hospital stay and a lower prevalence of hypertension, dyslipidaemia and active smoking. Diabetes mellitus was numerically less frequent, but the difference was not statistically significant. MINOCA more frequently presented as non-ST-elevation myocardial infarction (NSTEMI; 19 {51.4%} vs. 20 {13.9%}; p < 0.001) and was characterised by lower peak troponin T and CK-MB concentrations, higher left ventricular ejection fraction and a lower wall motion score index.

Conclusion: MINOCA should be approached as a heterogeneous working diagnosis rather than a uniform final diagnosis. The absence of obstructive coronary artery disease should prompt continued clinical evaluation and a structured, individualised diagnostic approach. Standardised diagnostic pathways and prospective multicentre studies incorporating comprehensive imaging and long-term clinical follow-up are needed to refine risk assessment and guide management.

## Introduction

Myocardial infarction (MI) represents one of the leading causes of morbidity and mortality worldwide, including in Europe and Bosnia and Herzegovina, where it remains the most frequent cause of death [1]. Traditionally, the pathophysiology of MI has been closely associated with obstructive coronary artery disease (CAD), and therapeutic strategies have been primarily focused on reperfusion and restoration of coronary blood flow. Early angiographic studies, such as those by DeWood and colleagues, demonstrated coronary occlusion in the vast majority of patients with acute MI, reinforcing the concept of obstructive lesions as the principal underlying mechanism [2]. However, subsequent registries and observational studies revealed that a notable proportion of patients fulfilling the diagnostic criteria for MI do not exhibit significant obstructive CAD on coronary angiography [3,4]. This led to the introduction of the concept of myocardial infarction with non-obstructive coronary arteries (MINOCA), nowadays recognised as a heterogeneous syndrome accounting for approximately 5-15% of all angiographically confirmed MI cases [5,6].

According to the Fourth Universal Definition of Myocardial Infarction developed by the Joint European Society of Cardiology/American College of Cardiology/American Heart Association/World Heart Federation (ESC/ACC/AHA/WHF) Task Force, acute MI requires evidence of acute myocardial injury accompanied by clinical evidence of acute myocardial ischaemia [7]. MINOCA is considered a working diagnosis when these criteria are fulfilled: coronary angiography demonstrates no obstructive lesion with stenosis ≥50%, and no alternative diagnosis fully explains the clinical presentation [7]. The underlying mechanisms are diverse and include plaque disruption without critical stenosis, spontaneous coronary artery dissection, epicardial or microvascular vasospasm, coronary thrombosis or embolism and coronary microvascular dysfunction [8-10]. Because of this heterogeneity, the clinical presentation may mimic both ST-elevation MI (STEMI) and non-ST-elevation MI (NSTEMI) and accurate diagnosis frequently requires a multimodal imaging approach, including echocardiography, cardiac magnetic resonance (CMR) and intracoronary imaging techniques [11]. Furthermore, while traditional cardiovascular risk factors are clearly linked to atherosclerotic MI, their role in the development of MINOCA has not been fully clarified [8-10]. Despite growing recognition of this entity, it is still underdiagnosed and important uncertainties remain regarding its natural history, risk profile and optimal therapeutic strategies.

Given the significant burden of MI in Bosnia and Herzegovina and the limited local data on MINOCA, this study had two aims. The primary aim was to determine the prevalence of MINOCA among patients with MI admitted to a tertiary cardiac centre in Bosnia and Herzegovina. The secondary aim was to compare the demographic characteristics, cardiovascular risk factors, electrocardiographic presentation, laboratory parameters and echocardiographic findings between an expanded cohort of patients with MINOCA and those with obstructive MI (OMI).

## Materials and methods

Subjects and study design

This retrospective single-centre observational study was conducted at the Clinic for Heart, Blood Vessel and Rheumatic Diseases, Clinical Centre University of Sarajevo, Bosnia and Herzegovina. The study comprised two complementary analyses: a prevalence analysis and a comparative analysis of patients with MINOCA and OMI.

For the prevalence analysis, medical records of patients admitted to the Intensive Cardiac Care Unit between April 2022 and February 2024 were reviewed. Among 2,341 admissions, 1,104 patients fulfilled the diagnostic criteria for MI, of whom thirty-two were diagnosed with MINOCA.

For the comparative analysis, an expanded cohort of 40 eligible patients with MINOCA identified between January 2020 and February 2024 was compared with 155 patients with OMI selected by simple random sampling from all eligible patients admitted with OMI during the same period (January 2020-February 2024), using a computer-generated random number sequence. Identical diagnostic criteria (the Fourth Universal Definition of Myocardial Infarction and the same MINOCA classification criteria) and the same case-ascertainment process were applied consistently throughout the 2020-2024 period covered by the comparative analysis.

MI was defined according to the Fourth Universal Definition of Myocardial Infarction developed by the Joint ESC/ACC/AHA/WHF Task Force [7]. The diagnosis required a rise and/or fall in cardiac troponin concentrations with at least one value above the 99th percentile upper reference limit. It also required at least one of the following: symptoms of acute myocardial ischaemia, new ischaemic electrocardiographic changes, development of pathological Q waves, imaging evidence of new loss of viable myocardium or a new regional wall motion abnormality consistent with an ischaemic aetiology or identification of a coronary thrombus.

Only adults aged 18 years or older who underwent diagnostic coronary angiography were eligible. Patients without coronary angiography, those with a previous MI and those who had undergone prior percutaneous coronary intervention or coronary artery bypass grafting were excluded.

MINOCA was considered a working diagnosis when the criteria for MI were fulfilled. Coronary angiography had to demonstrate no stenosis ≥50%, and no alternative diagnosis could adequately explain the clinical presentation. Patients with angiographically normal coronary arteries or stenosis <30%, as well as those with mild CAD with stenosis of 30% to <50%, were included in the MINOCA group. Advanced modalities (CMR, intracoronary imaging, dedicated myocarditis/Takotsubo work-up) were not routinely available and were not performed in any of the MINOCA patients (0%). Alternative diagnoses were therefore adjudicated clinically, based on presentation, ECG, biomarkers, angiographic and echocardiographic findings. 

The study was approved by the Institute for Scientific Research of the Clinical Centre of the University of Sarajevo (approval number: 51-30-5-15762/24). The study was conducted in accordance with the principles of the Declaration of Helsinki. Before analysis, all patient records were de-identified. Names, dates of birth, identification numbers and other direct personal identifiers were removed and replaced with unique study codes. 

Methods

Demographic characteristics, dates of admission and discharge, cardiovascular risk factors, smoking status, family history of CAD, electrocardiographic presentation, laboratory results, coronary angiographic findings and echocardiographic variables were extracted retrospectively from the patients’ medical records. Hypertension, diabetes mellitus and dyslipidaemia were considered present when a previous diagnosis was documented in the medical history or discharge record. Active smoking was defined as current tobacco use at the time of the index admission. A positive family history of CAD was defined as documented CAD in at least one first-degree relative. Electrocardiographic presentation was classified as STEMI or NSTEMI based on the admission electrocardiogram and the final clinical diagnosis. Length of hospital stay was calculated from the dates of admission and discharge.

For the laboratory analysis, peak troponin T, creatine kinase (CK), creatine kinase-MB (CK-MB) and lactate dehydrogenase (LDH) were defined as the highest recorded values during the index hospitalisation. Glucose was recorded from the admission laboratory assessment. Variables unavailable in the medical records were recorded as missing.

All patients underwent diagnostic coronary angiography. Based on the reported angiographic findings, patients were categorised as having obstructive CAD (stenosis ≥50%) or non-obstructive coronary artery disease (stenosis <50%). Patients with coronary stenosis <50% were assigned a working diagnosis of MINOCA only when they fulfilled the diagnostic criteria for MI, and no clinically evident alternative diagnosis was identified during the available in-hospital evaluation. Transthoracic echocardiography was performed in all patients using a GE Vivid S70N system (GE HealthCare, Chicago, IL, USA) equipped with an M5S-D phased-array transducer. Left ventricular ejection fraction (LVEF) and regional wall motion abnormalities were assessed. The wall motion score index (WMSI) was calculated using the standard 17-segment model. Each segment was scored as 1 (normal), 2 (hypokinetic), 3 (akinetic) or 4 (dyskinetic). The sum of the segmental scores was divided by the number of assessed segments to obtain the final WMSI [12]. Coronary angiograms and echocardiographic assessments were independently reviewed by two specialists each, blinded to group allocation, with disagreements resolved by consensus.

The primary outcomes were the prevalence of MINOCA among patients with MI in the prevalence cohort and the comparison of demographic characteristics and cardiovascular risk factors between patients with MINOCA and those with OMI. Secondary outcomes included between-group differences in electrocardiographic presentation, laboratory parameters and echocardiographic findings. No post-discharge follow-up was performed.

Statistical analysis

Statistical analysis was performed using IBM SPSS Statistics for Windows, Version 20.0 (IBM Corp., Armonk, NY, USA). The prevalence of MINOCA was calculated by dividing the number of patients with MINOCA by the total number of patients with MI in the prevalence cohort and was reported as a frequency and percentage.

The normality of continuous variables was assessed using the Shapiro-Wilk test. Categorical variables were reported as N (%), where N represents the number of patients with the characteristic of interest and the corresponding denominator for each variable is specified in the text or table footnotes where it differs from the total group size. Continuous variables were expressed as mean ± standard deviation for normally distributed data or as median and interquartile range for non-normally distributed data. Missing data were not imputed, and analyses were performed using available cases.

Between-group comparisons were performed using Pearson’s chi-square test for categorical variables, Student’s t-test for normally distributed continuous variables and the Mann-Whitney U test for non-normally distributed continuous variables. All statistical tests were two-sided, and p < 0.05 was considered statistically significant. All between-group comparisons were unadjusted; given the limited sample size of the MINOCA group, multivariable adjustment for potential confounders (e.g., age, sex) was not performed.

## Results

For the prevalence analysis, a total of 1,104 patients with MI were identified during the study period. Of these patients, 32 (2.9%) were diagnosed with MINOCA, and 1,072 (97.1%) had OMI.

The comparative analysis included 40 patients with MINOCA (20.5%) and 155 patients with OMI (79.5%). Patients with MINOCA (N = 40) were significantly younger than patients with OMI (N = 155) (52.1 ± 15.2 vs. 59.8 ± 11.7 years; p = 0.005).

Among patients with available sex data, female sex was more frequent in the MINOCA group (19 {47.5%}) than in the OMI group (43 {29.9%}; p = 0.037). The median length of hospital stay was significantly shorter in the MINOCA group (4 days; IQR 2-6) than in the OMI group (6 days; IQR 3-9; p < 0.001).

Hypertension was significantly less prevalent in the MINOCA group than in the OMI group (20 {50.0%} vs. 105 {68.6%}; p = 0.028). Dyslipidaemia was also less prevalent in the MINOCA group (12 {33.3%} vs. 86 {66.7%}; p < 0.001), as was active smoking (19 {47.5%} vs. 94 {64.8%}; p = 0.047). Diabetes mellitus was numerically less frequent in the MINOCA group, but the difference was not statistically significant (5 {13.5%} vs. 39 {28.1%}; p = 0.069). No statistically significant difference was observed in the prevalence of a positive family history of CAD (16 {69.6%} vs. 61 {58.1%}; p = 0.309) (Table 1).

**Table 1:** Demographic characteristics, cardiovascular risk factors and electrocardiographic presentation in patients with MINOCA and obstructive MI. MINOCA: myocardial infarction with non-obstructive coronary arteries; MI: myocardial infarction; CAD: coronary artery disease; SD: standard deviation; IQR: interquartile range; NSTEMI: non-ST-elevation myocardial infarction; STEMI: ST-elevation myocardial infarction. Categorical variables are presented as N (%). Percentages were calculated based on the number of patients with available data for each variable; therefore, denominators may differ from the total group sizes because of missing data. Continuous variables are presented as mean ± SD or median (IQR) as appropriate. Data were available for the following variables: female sex (MINOCA n=40, OMI n=144), hypertension (n=40, n=153), diabetes mellitus (n=37, n=139), dyslipidaemia (n=36, n=129), active smoking (n=40, n=145), family history of CAD (n=23, n=105), NSTEMI (n=37, n=144), STEMI (n=37, n=144).

Parameter	MINOCA (n=40)	Obstructive MI (n=155)	p-value
Female sex, N (%)	19 (47.5)	43 (29.9)	0.037
Age (years), mean ± SD	52.1 ± 15.2	59.8 ± 11.7	0.005
Hospital stay (days), median (IQR)	4 (2–6)	6 (3–9)	<0.001
Hypertension, N (%)	20 (50.0)	105 (68.6)	0.028
Diabetes mellitus, N (%)	5 (13.5)	39 (28.1)	0.069
Dyslipidaemia, N (%)	12 (33.3)	86 (66.7)	<0.001
Active smoking, N (%)	19 (47.5)	94 (64.8)	0.047
Family history of CAD, N (%)	16 (69.6)	61 (58.1)	0.309
NSTEMI, N (%)	19 (51.4)	20 (13.9)	<0.001
STEMI, N (%)	18 (48.6)	124 (86.1)	<0.001

Among patients with available electrocardiographic classification, NSTEMI was significantly more frequent in the MINOCA group (19 {51.4%}) than in the OMI group (20 {13.9%}; p < 0.001). Conversely, STEMI was significantly less frequent in the MINOCA group (18 {48.6%}) than in the OMI group (124 {86.1%}; p < 0.001) (Table 1).

Median peak troponin T was significantly lower in the MINOCA group than in the OMI group (356.0 ng/L; IQR 192.3-738.0 vs. 1,736.5 ng/L; IQR 618.8-3,641.8; p = 0.010). Median CK-MB was also significantly lower in the MINOCA group (85.0 U/L; IQR 43.8-141.3 vs. 151.0 U/L; IQR 57.5-331.0; p = 0.010). No statistically significant differences were observed in CK, LDH or admission glucose levels (Table 2).

**Table 2:** Laboratory parameters in patients with MINOCA and obstructive MI. MINOCA: myocardial infarction with non-obstructive coronary arteries; MI: myocardial infarction; CK: creatine kinase; CK-MB: creatine kinase-MB isoenzyme; LDH: lactate dehydrogenase; IQR: interquartile range. ' Data are presented as median (IQR). ^a^Data were available for 154 patients in the obstructive MI group. ^b^Data were available for 151 patients in the obstructive MI group.

Parameter	MINOCA (n=40)	Obstructive MI (n=155)	p-value
Peak troponin T (ng/L)	356.0 (192.3–738.0)	1736.5 (618.8–3641.8)	0.010
Peak CK (U/L)	448.5 (251.0–886.0)	992.0 (388.0–2126.5)^a^	0.120
Peak CK-MB (U/L)	85.0 (43.8–141.3)	151.0 (57.5–331.0)^a^	0.010
Peak LDH (U/L)	305.0 (215.0–392.0)	396.5 (247.0–696.0)^a^	0.285
Admission glucose (mmol/L)	6.75 (5.56–8.25)	7.15 (5.93–9.86)^b^	0.971

Median left ventricular ejection fraction was significantly higher in the MINOCA group (N = 40; 51.0%; IQR 48.5-62.0) than in the OMI group (N = 155; 42.4%; IQR 40.0-55.0; p < 0.001). Mean WMSI was significantly lower in the MINOCA group (1.20 ± 0.26 vs. 1.40 ± 0.31; p < 0.001) (Table 3).

**Table 3:** Echocardiographic parameters in patients with MINOCA and obstructive MI. MINOCA: myocardial infarction with non-obstructive coronary arteries; MI: myocardial infarction; LVEF: left ventricular ejection fraction; WMSI: wall motion score index; IQR: interquartile range; SD: standard deviation. Data are presented as median (IQR) or mean ± SD as appropriate.

Parameter	MINOCA (n=40)	Obstructive MI (n=155)	p-value
LVEF (%), median (IQR)	51.0 (48.5–62.0)	42.4 (40.0–55.0)	<0.001
WMSI, mean ± SD	1.20 ± 0.26	1.40 ± 0.31	<0.001

## Discussion

This study evaluated the prevalence and clinical characteristics of MINOCA at a tertiary cardiac centre in Bosnia and Herzegovina and compared them with those of patients with OMI. MINOCA accounted for 2.9% of MI cases included in the prevalence analysis. In the comparative analysis, patients with MINOCA were significantly younger, a higher proportion were women, and the median length of hospital stay was significantly shorter. Hypertension, dyslipidaemia and active smoking were significantly less prevalent in the MINOCA group, while diabetes mellitus was numerically less frequent without reaching statistical significance. Patients with MINOCA had lower peak troponin T and CK-MB concentrations, higher LVEF, a lower WMSI and a more frequent NSTEMI presentation than patients with OMI.

The prevalence of 2.9% observed in this study is comparable with earlier reports. Larsen et al. reported a prevalence of 2.8% among patients with angiographically normal coronary arteries [4]. Other studies have reported higher rates, with Chen et al. reporting 3.4%, Dreyer et al. 5.9% and Bossard et al. 6.7% [13-15]. Differences between studies may reflect variation in the populations examined, definitions applied, use of coronary angiography and methods used to exclude alternative diagnoses. Data from the same centre also illustrate this variability, as Sokolović et al. reported a prevalence of 0.7% between 2018 and 2019 [16]. The higher proportion identified in the present study may partly reflect improved recognition of MINOCA and greater adherence to contemporary diagnostic definitions. Moreover, the absence of systematic CMR imaging means that myocarditis, Takotsubo syndrome and other alternative diagnoses may not have been uniformly excluded and could have influenced the estimated prevalence. The prevalence estimate of 2.9% reflects our specific study population and timeframe and may differ in other settings.

Given the significant baseline differences in age and sex between the MINOCA and OMI groups, the observed differences in cardiovascular risk factors, hospital stay, laboratory biomarkers, LVEF and WMSI cannot necessarily be attributed independently to MINOCA status and may, at least in part, reflect these underlying baseline imbalances. A multivariable adjusted analysis was considered; however, given the limited sample size of the MINOCA group (n = 40), such an analysis would have been underpowered and prone to overfitting, and was therefore not performed. Accordingly, the reported associations should be interpreted as unadjusted descriptive findings. Additionally, the exact size of the source population of eligible OMI patients from which the comparative cohort was randomly selected was not retained, which limits full transparency regarding the sampling frame.

The demographic profile observed in this study is broadly consistent with previous reports. Pasupathy et al. reported that women accounted for approximately 43% of MINOCA cases compared with 24% of patients with obstructive myocardial infarction [6]. The reported mean age of patients with MINOCA was 54.7 years, which was also comparable with the present cohort [6]. However, some single-centre studies have reported different distributions. Sokolović et al. observed a predominance of men, while Chen et al. reported that men represented 71.9% of the MINOCA population [13,16]. These differences may reflect regional variation in patient populations, referral patterns, case selection and diagnostic practices.

The shorter hospital stay among patients with MINOCA is consistent with the findings of Haertel et al. [17]. This difference may reflect less extensive myocardial injury and fewer early complications. Several potential mechanisms of MINOCA involve transient ischaemia, coronary spasm, microvascular dysfunction or limited plaque disruption rather than persistent epicardial coronary occlusion. These mechanisms could result in more limited myocardial injury. Nevertheless, differences in local clinical practice and discharge criteria may also have influenced the duration of hospitalisation.

Hypertension, dyslipidaemia and active smoking were significantly less prevalent in the MINOCA group. Diabetes mellitus was also less frequent, but the difference was not statistically significant. No significant difference was observed in family history of CAD. Previous studies have reported variable findings regarding traditional cardiovascular risk factors. Lopez-Pais et al. identified active smoking as the only significantly different risk factor, while Pasupathy et al. identified dyslipidaemia as the only significantly different factor [6,18]. Karaca et al. reported significant differences in family history, diabetes mellitus and smoking status [19]. This variability is consistent with the heterogeneous mechanisms underlying MINOCA, which may include coronary spasm, spontaneous coronary artery dissection, coronary thromboembolism and microvascular dysfunction. However, the present findings do not establish that traditional atherosclerotic risk factors are unimportant in MINOCA.

Lower peak troponin T and CK-MB concentrations in the MINOCA group are consistent with observations by Lopez-Pais et al. and Hjort et al. [18,20]. These findings may reflect a smaller extent of myocardial injury, although infarct size was not directly assessed in the present study. Higher LVEF and a lower WMSI may similarly indicate more limited myocardial involvement. These findings are consistent with the meta-analysis by Pizzi et al., which reported an average LVEF of approximately 50% among patients with MINOCA [21]. Taken together, these observations support the possibility of more localised or transient ischaemic injury in this population. However, cardiac biomarker concentrations may also be influenced by the timing of measurement and other clinical factors.

NSTEMI presentation was significantly more frequent among patients with MINOCA. This finding is consistent with the meta-analysis by Pasupathy et al. and the RENASCA study by Borrayo-Sánchez et al. [6,22]. Potential explanations include transient coronary occlusion, microvascular dysfunction, coronary spasm or limited plaque disruption, which may be less likely to produce persistent transmural ischaemia and ST-segment elevation. The present study did not include the advanced diagnostic evaluation required to determine which of these mechanisms was responsible in individual patients.

This study adds local epidemiological and clinical data on MINOCA from Bosnia and Herzegovina, where detailed information regarding this condition remains limited. The findings highlight the importance of applying contemporary diagnostic criteria and maintaining clinical awareness of MINOCA as a working diagnosis. They also support the need for a structured diagnostic approach aimed at identifying the underlying mechanism rather than treating MINOCA as a single disease entity.

Several limitations should be acknowledged. The retrospective single-centre design introduces the generalisability of the findings which, potentially, could limit interpretation. The number of patients with MINOCA was relatively small, and sample sizes varied across analyses because of missing data. As none of the 40 patients classified as MINOCA (0%) underwent CMR or other advanced diagnostic modalities, the reported prevalence of MINOCA should be interpreted with caution, as it may be affected by misclassification of non-ischaemic causes of myocardial injury that were not systematically excluded. No post-discharge follow-up was performed, which prevented assessment of long-term clinical outcomes. Future prospective multicentre studies incorporating multimodal imaging, multivariable analyses and standardised follow-up are needed to better characterise the mechanisms, clinical course and individualised treatment requirements of patients with MINOCA.

## Conclusions

MINOCA should be approached as a heterogeneous working diagnosis rather than a uniform final diagnosis. In a patient who meets the diagnostic criteria for MI, the absence of obstructive CAD should prompt continued clinical evaluation rather than represent the endpoint of the diagnostic process. A structured and individualised approach can help clinicians integrate the available clinical, laboratory and imaging findings and support appropriate diagnostic and management decisions.

Greater consistency in documenting and evaluating suspected MINOCA may improve diagnostic clarity and strengthen the quality of local clinical data in Bosnia and Herzegovina. Future prospective multicentre studies incorporating comprehensive cardiac and coronary imaging and long-term clinical follow-up are needed to refine diagnostic pathways and guide management within different healthcare resource settings.
